# A Fast-Neutron Diagnostic Probe

**DOI:** 10.6028/jres.093.127

**Published:** 1988-06-01

**Authors:** C. M. Gordon, C. W. Peters, T. K. Olson

**Affiliations:** Consolidated Controls Corporation, Lockport Place, Lorton, VA 22079

A novel, nuclear method for instrumental analysis of elements has been developed. The system, called a “neutron diagnostic probe (NDP),” has several unusual capabilities that are especially applicable to nondestructive, 3-dimensional analysis in solid materials or inaccessible spaces. The prototype instruments that have been assembled and tested demonstrate that the NDP system can penetrate several inches of solids, locate inaccessible inhomogeneities by time-of-flight ranging, and perform elemental analysis of the located volume of interest. This neutron diagnostic probe utilizes a combination of associated-particle, neutron time-of-flight spectroscopy; inelastic gamma-ray spectroscopy; and a recently developed sealed-tube neutron generator. The NDP interrogates a material or inaccessible space with an electronically collimated and timed beam of fast neutrons which have great penetrating power. Highly penetrating, inelastic gamma rays produced in the interrogated volume by the neutrons are detected and analyzed. The spatial production of these gamma rays is analyzed to “image” the distribution of materials within the volume of interest. The energies of the gamma rays from each substance within the space examined are analyzed to determine the concentrations of the elements in each volume element. An inelastic gamma-ray energy spectrum characteristic of every element except hydrogen can be obtained.

The name of this associated-particle technique [1] is derived from the alpha particle “associated” with a 14-MeV neutron produced by the T(d,n)He^4^ reaction in the neutron generator. Because the associated particle is produced simultaneously with the fast neutron and is emitted in the opposite direction, its detection specifies the subsequent trajectory of the individual neutron. This capability makes it possible to define a timed and directed “beam” of 14-MeV neutrons traveling away from a continuous T(d,n)He^4^ generator at about 5 cm per nanosecond. By using time-of-flight, range gating, only gamma rays generated by neutron reactions within a known sensitive volume at a predetermined distance from the generator are recorded.

The key element in the instrumentation is the sealed-tube neutron generator (STNG) shown schematically in figure 1. The basic design of the neutron generator is similar to that described by Reifenschweiler [2,3], using the T(d,n)He^4^ reaction, a getter-controllable mixture of deuterium and tritium gases and a self-loading target. The innovations in the STNG used for this work are: 1) the inclusion of an internal alpha detector to supply time and direction information by the associated-particle technique and 2) provision for focusing the ion beam on the target to ensure a “point source” of 14-MeV neutrons.

In operation, the radiation detectors and nuclear electronics are arranged as shown in the block diagram of figure 2. Light signals from the ZnS alpha scintillator are amplified by a photomultiplier (PM) and preamplifier, shaped by a constant-fraction discriminator (CFD) and routed to the “start” input of a time-to-amplitude converter (TAC). The alpha signals are also counted and stored in the multichannel analyzer (MCA) data acquisition system for subsequent normalization of the spectral information. The gamma rays generated by inelastic neutron interactions in the interrogated sample are converted to energy spectra with a 15 cm diameter by 36 cm long NaI(T1) scintillation spectrometer. These signals are shaped (CFD) and routed to the “stop” input of the TAC to provide a range-proportional, time-of-flight spectrum at the TAC output. The time-of-flight spectrum is proportional to range because the 14-MeV neutrons travel from the STNG to the interrogated sample at a constant 5 cm/ns and the gamma rays produced in the sample travel at 30 cm/ns to the NaI(T1) detector. Signals from the portion of the time-of-flight spectrum that corresponds to the range position of the sample are selected to open a gate circuit (linear gate) and pass only gamma-ray signals that occur within the appropriate delayed-coincidence, time gate.

The gamma-ray pulses are also linearly amplified and because of the time gate (range gate) only those analog signals of gamma rays originating in the selected sample volume are digitally converted (ADC) and recorded in the data acquisition system. This range-gating technique (time gate of about 3 ns) eliminates nearly all background. In general the background count rate (*N*_c_) is expressed as

$$N_{c}=\tau N_{\alpha }N_{\gamma },$$

where *τ* is the duration of the time gate while N*_α_* and N*_γ_* are the random count rates in the alpha detector and gamma detector respectively. In the system described here N_α_≈3×10^3^ s^−1^ and *τ*≈3×10^−9^ s, therefore a random rate N*_γ_*≈1×10^4^ s^−1^ in the gamma detector only generates *N*_c_≈10^−1^ s^−1^ background counts. As with all nuclear methods, the ultimate precision of the measurements will depend on signal-to-noise ratio and counting statistics, i.e., a standard deviation of approximately $\pm \sqrt{N}$ where *N* is the total count. In circumstances requiring background subtractions or other data manipulations the usual rules for propagation of precision indices apply.

In an application that might be called “remote trace analysis” the timed beam of 14-MeV neutrons was directed through about 1 inch of steel to monitor Na_2_SO_4_ corrosive agent on turbine blades. Measurements at less than 1/10,000 Na were made. Thus far the neutron diagnostic probe has been used mostly in areas of bulk analysis of oil shale, coal and sandstone and detection of concealed explosives or contraband, however it is expected to find additional applications for special-purpose instrumental trace analysis where requirements of nondestructive imaging and inaccessibility are paramount.

## Figures and Tables

**Figure 1:**
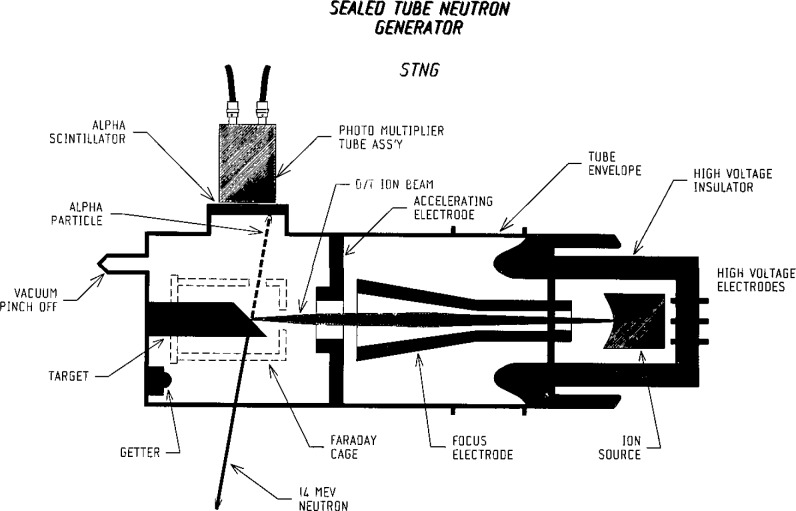
A schematic diagram of the neutron generator.

**Figure 2:**
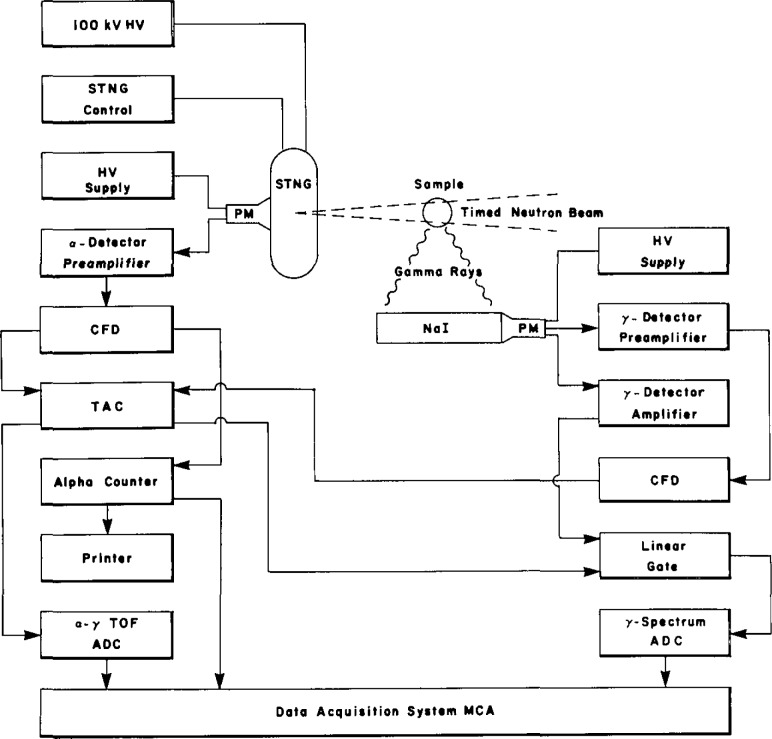
A block diagram of the experimental arrangement.
